# Inequities in the Application of Behavioral Flags for Hospitalized Pediatric Patients

**DOI:** 10.1001/jamanetworkopen.2024.61079

**Published:** 2025-02-20

**Authors:** April Edwell, Jia Xin Huang, Tasce Bongiovanni, Matthew Pantell

**Affiliations:** 1 Department of Pediatrics, University of California, San Francisco; 2 Department of Surgery, University of California, San Francisco

## Abstract

**Importance:**

Behavioral flags in the electronic health record (EHR) may introduce bias and perpetuate structural racism and discrimination. Descriptions of differences in the way that markers of behavioral risk are communicated will help clarify the inequities that pediatric patients and their families experience in the hospital.

**Objective:**

To assess whether racially and socioeconomically marginalized pediatric patients and families are more likely than their counterparts to be assigned a behavioral flag in their EHR.

**Design, Setting, and Participants:**

This retrospective cohort study used EHR data for pediatric patients (aged <18 years) hospitalized from June 2012 to July 2021 across care settings at the University of California, San Francisco health care facilities, an academic quaternary care hospital system that includes 2 pediatric inpatient facilities. The analysis was completed from December 29, 2022, to November 22, 2024.

**Main Outcome and Measures:**

The primary outcome of interest was any of the following behavioral flags placed in a patient’s EHR: witnessed substance abuse, history of inappropriate behavior, security, violent behavior, dismissal from practice, and child protective services (CPS) hold. The primary variables were patients’ race, ethnicity, insurance status, and primary language.

**Results:**

Of 55 865 pediatric encounters (52.2% among males; median patient age at the first encounter, 3 years [IQR, 0-12 years]), 236 (0.4%) had behavioral flags. Compared with encounters among patients who identified as White, encounters among patients who identified as Black or African American were more likely to have a behavioral flag (incidence rate ratio [IRR], 2.07; 95% CI, 1.32-3.25). Behavioral flags were also more likely among encounters of individuals with government insurance compared with those with private insurance (IRR, 2.60; 95% CI, 1.85-3.65). Black or African American patients younger than 1 year (IRR, 3.53; 95% CI, 1.80-6.91) and aged 1 to 7 years (IRR, 2.87; 95% CI, 1.34-6.15) had a higher likelihood of flag placement compared with their White counterparts.

**Conclusions and Relevance:**

This cohort study found significant inequities in incidence of behavioral flags in the EHR among racially and socioeconomically marginalized pediatric patients. This finding was most pronounced for Black or African American patients younger than 8 years, suggesting that this phenomenon may be a response to Black families rather than specific patient behavior.

## Key Points

**Question:**

Are there demographic differences in application of behavioral flags to pediatric patients’ electronic health records (EHRs)?

**Findings:**

In this cohort study of 55 865 pediatric patient encounters, Black or African American patients, particularly those younger than 8 years, had increased incidence rate ratios of behavioral flags placed in their EHRs compared with White patients. Behavioral flags were also more commonly assigned among individuals with government vs private insurance.

**Meaning:**

These findings suggest that flag application may be a response to Black families rather than patients alone.

## Teaser

This cohort study among pediatric patients in an urban academic health system examines whether receiving a behavioral flag in the electronic health record is associated with racial, ethnic, and socioeconomic variables.

## Introduction

Pervasive inequities in health and health care outcomes exist based on race, ethnicity, and socioeconomic status. These inequities are due in part to structural racism and discrimination (SRD),^1,2^ which the National Institute on Minority Health and Health Disparities defines as “macro-level conditions (eg, residential segregation and institutional policies) that limit opportunities, resources, power, and well-being of individuals based on race and ethnicity and other statuses.”^2^ Much prior work has focused on the downstream health consequences of SRD.^3,4,5,6,7^ For example, many studies have shown how neighborhood segregation contributes to adverse health outcomes such as preterm birth, and more recently, numerous studies have linked SRD to the inequities in mortality from the COVID-19 pandemic.^3,8,9,10,11^ Fewer studies have examined inequities in more proximal actions that may be manifestations of SRD in health care settings, such as behavioral flags, the labeling of patients as having a concerning or threatening behavioral characteristic in electronic health records (EHRs). Prior work has shown that Black patients have 2.5-times increased odds of having negative descriptors in their medical notes compared with White patients, which raises concern that there may be biases in information documented in other parts of the EHR.^12^

Behavioral flags were initially developed to identify and track patients at high risk of violent behavior.^13^ This paralleled other efforts by health care systems and states to reduce workplace violence, such as California’s 1995 Hospital Safety and Security Act, requiring hospitals to report violent events.^14^ While some studies^15,16^ have found flags to be “reasonably effective in identifying potentially violent or aggressive patients,”^16^ a Cochrane review concluded that most risk interventions (including flags) do not reliably lead to a reduction in violent incidents.^16^ These flags may be well intentioned at times (eg, to inform staff that a patient has previously been physically violent in a health care setting). However, behavioral flags have the potential to cause harm to patients if applied disparately based on patient characteristics, particularly by perpetuating SRD. EHRs have been implicated in inequitable health outcomes; if there is bias in information entered into the EHR, subsequent users can be influenced by this information, ultimately contributing to inequitable patient outcomes.^17^ Prior work has found that Black patients who received flags were also subject to longer wait times and were less likely to get laboratory testing and imaging compared with their White counterparts.^18^

However, there has been little research into potential inequities in behavioral flag application across hospital settings. Studies of adults, specifically in the emergency department setting, have found that Black patients received behavioral flags at higher rates than White patients, and there is research showing that there are racial and ethnic disparities in inpatient security events.^18,19,20,21^ In our own hospital system, a study found that Black newborns and mothers were being screened more often for drugs in the intensive care nursery.^22^

To our knowledge, no studies have analyzed behavioral flags in the pediatric inpatient setting. Investigating the patterns and trends of receiving behavioral flags for children and their families will not only shed light on the treatment of this vulnerable group but may also hold clues to how and why stigma is attached within the health care system and continues with these patients. Therefore, the objective of this study was to examine demographic variables associated with receipt of a behavioral flag for pediatric patients in our academic health center.

## Methods

### Study Design and Setting

We conducted a retrospective cohort study of pediatric patients hospitalized at the University of California, San Francisco (UCSF) health care facilities, an urban, quaternary academic health system with 2 pediatric inpatient facilities, from June 2012 to July 2021. All data were extracted from structured fields in the EHR by the UCSF Clinical & Translational Science Institute. This study followed the Strengthening the Reporting of Observational Studies in Epidemiology (STROBE) reporting guideline.^23^ The study was approved by the UCSF institutional review board, which waived informed consent because all data were fully deidentified and the study posed minimal risk to participants.

### Variable Definitions

The primary outcome was the presence of any of the following predefined behavioral flags placed in a patient’s EHR: witnessed substance abuse, history of inappropriate behavior, security, violent behavior, dismissal from practice, and child protective services (CPS) hold. In our hospital system, any staff member can escalate a behavioral concern to the workplace violence prevention team and safety office, which then determines whether and what kind of a behavioral flag should be placed in the EHR. Once the flag is placed, subsequent entries into the patient’s EHR trigger a pop-up window with information about the flag that must be closed before navigating the patient’s EHR. During the study period, there were no guidelines on what warranted a flag being placed or when the flags would be removed. It was also unclear how the placement of a flag influenced practitioners’ actions after it was placed (eg, we could not distinguish when hospital security or police were physically present or whether or when restraints or force were used based on the presence of behavioral flags alone) and whether the flag was intended for the patient or for caregivers. We analyzed the presence of any behavioral flag as well as the flag type.

The primary variables were race, ethnicity, insurance status, and primary language. Race and ethnicity data were based on patient or caregiver self-report during the time of registration per our health system’s predefined categories. Race categories included American Indian or Alaska Native, Asian, Black or African American, Native Hawaiian or Other Pacific Islander, White, other (unspecified race), or unknown or declined (if the information was not available). Ethnicity was categorized into Hispanic or Latino, not Hispanic or Latino, or unknown or declined. Preferred language, based on patient or caregiver report, was collapsed into 5 categories to include the 3 most common languages—Chinese (Cantonese, Hsiang, Mandarin, Mien, Toishanese, or other dialect), English, and Spanish—as well as other and unknown. Types of insurance were characterized as government or public, private (all other types), or unknown or declined. Insurance was used as a marker of socioeconomic status.

Covariates included age and sex. We categorized age into groups of younger than 1 year, 1 to 7 years, 8 to 12 years, and 13 to 17 years to reflect different developmental stages of pediatric patients. Sex was categorized as female, male, or unknown. There were 16 individuals whose sex was nonbinary in the EHR, which may have been miscoding of gender as sex; thus, those individuals were removed from the analysis.

A formal sample size calculation was not performed before the study. To maximize the sample size, we included all encounters with patients aged 0 to 17 years with any flag placed within the study period.

### Statistical Analysis

We calculated frequencies and percentages of the outcome and variables of interest. Rates of having any behavioral flag were compared using Pearson χ^2^ tests. We calculated incident rate ratios (IRRs) of having behavioral flags by fitting a negative binomial regression model using individual variables, accounting for the number of days hospitalized in the regression model. We chose to use negative binomial regression to model the distribution characteristics of count data. We calculated IRRs using 2 models: (1) a partially adjusted model with only 1 variable controlling for age and sex and (2) a fully adjusted, saturated model including all variables in addition to age and sex. Language and insurance other than government or private were not included in the fully adjusted, saturated model due to the low number of participants in these categories. We chose not to combine racial groups in our analysis to preserve the specific racial categories recorded in the EHR, as combining them would make it difficult to interpret results meaningfully. White race and non-Hispanic ethnicity were chosen as the reference groups to increase precision of estimates because they were the largest groups. We attempted to perform the same negative binomial regression analyses to assess each individual type of behavioral flag but limited this analysis to the 2 flag types with the largest sample size, which were security and CPS hold. We analyzed the IRRs for all types of flags placed across racial groups specifically and conducted a stratified analysis by age group (<1 year, 1-7 years, 8-12 years, and 13-17 years) to identify flagging patterns within specific racial and age categories. This approach allowed us to assess whether flags were likely applied due to actions by caregivers rather than the patients themselves, particularly for younger age groups, such as infants, who are less likely to engage in behaviors warranting such flags.

We performed an encounter-level analysis, whereby all encounters were included in the analysis (ie, 1 patient could have multiple encounters). To account for encounters with behavioral flags influencing behavioral flag use in subsequent encounters, we also performed a sensitivity analysis including only the first encounter for each patient for those hospitalized multiple times. We performed an additional sensitivity analysis of the fully adjusted saturated model limited to infants younger than 1 year to further evaluate whether placement of flags was more related to caregiver actions than to behaviors by the patients themselves. We performed all analyses from December 29, 2022, to November 22, 2024, using Stata, version 16.1 (StataCorp LLC). Two-sided *P* <.05 was considered significant.

## Results

This cohort study included 55 865 pediatric encounters over 9 years. Median patient age at the first encounter was 3 years (IQR, 0-12 years). Of all encounters, 47.8% were among females; 52.2%, among males; and less than 1%, among patients with unknown sex. A total of 1.2% were among American Indian or Alaska Native patients; 12.1%, among Asian patients; 8.4%, among Black or African American patients; 1.2%, among Native Hawaiian or Other Pacific Islander patients; 39.0%, among White patients; 34.0%, among patients reporting other race; and 4.2%, among those with unknown or declined race. Overall, 34.5% of encounters were among Hispanic or Latino patients; 60.9%, among non-Hispanic or non-Latino patients; and 4.6%, among those with unknown ethnicity. Patients with government insurance accounted for 53.5% of encounters. We observed a total of 236 behavioral flags (0.4%) across all encounters, with most flags placed in encounters of patients younger than 8 years (143 [60.6%]) and, in particular, younger than 1 year (88 [37.3%]); White patients (77 [32.6%]); non-Hispanic or non-Latino patients (153 [64.8%]); and patients with government insurance (189 [80.1%]) (Table 1). The most common flag types were for security (105 [44.5%]) followed by CPS hold (80 [33.9%]), violent behavior (28 [11.9%]), and inappropriate behavior (21 [8.9%]) (Table 2). There were significant differences in the frequency of flags placed across racial groups for inappropriate behavior, security, violent behavior, and CPS hold.

**Table 1.** Demographic and Encounter Characteristics by Presence of a Flag for All Encounters

Characteristic	Participants, No. (%)			*P* value
	Total (N = 55 865)	Flag (n = 236)	No flag (n = 55 629)	
Age, y				
<1	14 991 (26.8)	88 (37.3)	14 903 (26.8)	.002
1-7	17 763 (31.8)	55 (23.3)	17 707 (31.8)	
8-12	9606 (17.2)	40 (17.0)	9555 (17.2)	
13-17	13 505 (24.2)	53 (22.5)	13 448 (24.2)	
Sex				
Female	26 676 (47.8)	120 (50.9)	26 556 (47.8)	.63
Male	29 168 (52.2)	116 (49.2)	29 052 (52.2)	
Unknown	5 (<0.1)	0	5 (<0.1)	
Race				
American Indian or Alaska Native	681 (1.2)	9 (3.8)	672 (1.2)	<.001
Asian	6747 (12.1)	14 (5.9)	6733 (12.1)	
Black or African American	4676 (8.4)	59 (25.0)	4617 (8.3)	
Native Hawaiian or Other Pacific Islander	647 (1.2)	4 (1.7)	643 (1.2)	
White	21 805 (39.0)	77 (32.6)	21 721 (39.1)	
Other^a^	18 991 (34.0)	55 (23.3)	18 927 (34.0)	
Unknown or declined	2318 (4.2)	18 (7.6)	2300 (4.1)	
Ethnicity				
Hispanic or Latino	19 267 (34.5)	66 (28.0)	19 200 (34.5)	.03
Not Hispanic or Latino	34 018 (60.9)	153 (64.8)	33 850 (60.9)	
Unknown	2580 (4.6)	17 (7.2)	2563 (4.6)	
Language				
English	47 304 (84.7)	228 (96.6)	47 060 (84.6)	<.001
Spanish	7074 (12.7)	7 (3.0)	1067 (12.7)	
Chinese	448 (0.8)	0	448 (0.8)	
Other	887 (1.6)	1 (0.4)	886 (1.6)	
Unknown or declined	152 (0.3)	0	152 (0.3)	
Insurance				
Government	20 906 (53.5)	189 (80.1)	29 708 (53.4)	<.001
Private	25 345 (45.4)	47 (19.9)	25 298 (45.5)	
Other	614 (1.1)	0	607 (1.1)	

^a^ Race was unspecified.

**Table 2.** Types of Flags by Racial Groups for All Encounters

Type of flag	**Encounters with a flag, No. (%)**								*P* value
	Total (N = 236)	American Indian or Alaska Native (n = 9)	Asian (n = 14)	Black or African American (n = 59)	Native Hawaiian or Other Pacific Islander (n = 4)	White (n = 77)	Other (n = 49)	Unknown or declined (n = 24)	
Inappropriate behavior	21 (8.9)	0	1 (7.1)	6 (10.2)	2 (50.0)	10 (13.0)	2 (4.1)	0	<.001
Security	105 (44.5)	6 (66.7)	3 (21.4)	20 (33.9)	1 (25.0)	38 (49.4)	30 (61.2)	7 (29.2)	<.001
Witnessed substance use	2 (0.8)	0	0	0	0	2 (2.6)	0	0	.79
Violent behavior	28 (11.9)	0	8 (57.1)	8 (13.6)	0	6 (7.8)	6 (12.2)	0	<.001
CPS hold	80 (33.9)	3 (33.3)	2 (14.3)	25 (42.4)	1 (25.0)	21 (27.3)	11 (22.4)	17 (70.8)	<.001

Abbreviation: CPS, child protective services.

Our analysis identified racial and socioeconomic disparities in flag placements. Encounters for Black or African American patients had a higher incidence of flags placed compared with those for White patients (IRR, 2.07; 95% CI, 1.32-3.25) (Table 3). Encounters for patients with public insurance had higher likelihood of having a flag placed compared with those with private insurance (IRR, 2.60; 95% CI, 1.85-3.65).

**Table 3.** Flag IRR by Race, Gender, and Insurance Among All Patients Younger Than 18 Years for All Encounters

Variables	Unadjusted		Adjusted^a^	
	IRR (95% CI)	*P* value	IRR (95% CI)	*P* value
Race				
American Indian or Alaska Native	2.51 (0.94-6.71)	.07	2.05 (0.76-5.53)	.16
Asian	1.02 (0.65-1.62)	.92	1.28 (0.81-2.04)	.29
Black or African American	2.90 (1.88-4.47)	<.001	2.07 (1.32-3.25)	.002
Native Hawaiian or Other Pacific Islander	1.06 (0.29-3.97)	.93	0.87 (0.23-3.23)	.84
White	1 [Reference]	NA	1 [Reference]	NA
Other^b^	0.71 (0.49-1.03)	.07	0.55 (0.36-0.85)	.007
Unknown or declined	2.27 (1.24-4.15)	.008	2.53 (1.04-6.16)	.04
Ethnicity				
Hispanic or Latino	0.72 (0.52-0.99)	.40	0.94 (0.63-1.40)	.76
Not Hispanic or Latino	1 [Reference]	NA	1 [Reference]	NA
Unknown or declined	1.30 (0.71-2.40)	.40	0.73 (0.29-1.85)	.51
Insurance				
Private	1 [Reference]	NA	1 [Reference]	NA
Government	2.38 (1.75-3.25)	<.001	2.60 (1.85-3.65)	<.001

Abbreviations: IRR, incident rate ratio; NA, not applicable.

^a^ Adjusted for the following variables except for the variable of analysis: race, ethnicity, and insurance status.

^b^ Race was unspecified.

When examining flag types, we found that patients with government insurance were more likely to have a security flag placed compared with those with private insurance (IRR, 3.23; 95% CI, 1.98-5.27) (Table 4). CPS flags were also more common among encounters for Black or African American patients (IRR, 2.25; 95% CI, 1.21-4.20) compared with White patients and for patients with government insurance (IRR, 5.79; 95% CI, 2.94-11.43) compared with private insurance.

**Table 4.** IRR for Security and CPS Hold Flags by Race, Sex, and Insurance for All Encounters^a^

Type of flag, variable	Unadjusted		Adjusted^b^	
	IRR (95% CI)	*P* value	IRR (95% CI)	*P* value
**Security**				
Race				
American Indian or Alaska Native	3.64 (1.32-10.02)	.01	2.70 (0.97-7.45)	.06
Asian	0.20 (0.06-0.66)	.01	0.21 (0.06-0.69)	.01
Black or African American	2.24 (1.30-3.88)	.004	1.52 (0.86-2.68)	.15
Native Hawaiian or Other Pacific Islander	0.48 (0.06-4.05)	.50	0.39 (0.04-3.37)	.39
White	1 [Reference]	NA	1 [Reference]	NA
Other^c^	0.90 (0.57-1.42)	.65	0.67 (0.38-1.16)	.16
Unknown or declined	1.82 (0.81-4.08)	.15	1.44 (0.45-4.64)	.54
Ethnicity				
Not Hispanic or Latino	1 [Reference]	NA	1 [Reference]	NA
Hispanic or Latino	1.03 (0.69-1.54)	.90	0.93 (0.55-1.56)	.77
Unknown or declined	1.57 (0.71-3.45)	.26	1.20 (0.38-3.82)	.76
Insurance				
Private	1 [Reference]	NA	1 [Reference]	NA
Government	3.31 (2.08-5.26)	<.001	3.23 (1.98-5.27)	<.001
**CPS hold**				
Race				
American Indian or Alaska Native	1.94 (0.45-8.34)	.37	1.27 (0.31-5.28)	.74
Asian	0.16 (0.04-0.71)	.02	0.20 (0.04-0.88)	.03
Black or African American	3.45 (1.88-6.30)	<.001	2.25 (1.21-4.20)	.01
Native Hawaiian or Other Pacific Islander	0.82 (0.09-7.28)	.86	0.68 (0.08-5.94)	.73
White	1 [Reference]	NA	1 [Reference]	NA
Other^c^	0.56 (0.30-1.03)	.06	0.32 (0.16-0.64)	.001
Unknown or declined	3.44 (1.63-7.25)	.001	3.35 (1.11-10.12)	.03
Ethnicity				
Not Hispanic or Latino	1 [Reference]	NA	1 [Reference]	NA
Hispanic or Latino	0.94 (0.57-1.53)	.79	1.30 (0.71-2.38)	.39
Unknown or declined	2.28 (1.03-5.06)	.04	0.86 (0.25-2.90)	.80
Insurance				
Private	1 [Reference]	NA	1 [Reference]	NA
Government	6.20 (3.21-11.98)	<.001	5.79 (2.94-11.43)	<.001

Abbreviations: CPS, child protective services; NA, not applicable; IRR, incident rate ratio.

^a^ IRRs for the other types of flags (inappropriate behavior, witnessed substance use, and violent behavior) are not shown due to small sample size and inability to calculate the IRR.

^b^ Adjusted for the following variables except for the variable of analysis: race, ethnicity, and insurance status.

^c^ Race was unspecified.

Stratified analysis of flag placement by age and racial group revealed age-specific disparities, with Black or African American patients younger than 1 year (IRR, 3.53; 95% CI, 1.80-6.91) and aged 1 to 7 years (IRR, 2.87; 95% CI, 1.34-6.15) having higher likelihood of flag placement compared with White patients in the same age groups (Table 5). Among those aged 8 to 12 years, American Indian or Alaska Native (IRR, 8.75; 95% CI, 1.21-63.21) and Asian (IRR, 5.60; 95% CI, 2.26-13.92) patients had the highest likelihood of having a flag placed compared with White children. Among teenagers (age 13-17 years), those with an unknown or declined racial category in their EHR were more likely to have a flag placed compared with White teenagers (IRR, 4.25; 95% CI, 1.28-14.15). We further evaluated the likelihood of flags being placed in encounters for infants (age <1 year) after adjusting for race and insurance and found that compared with White patients, Black or African American patients (IRR, 1.96; 95% CI, 1.00-3.82) and those whose race was unknown or declined (IRR, 3.97; 95% CI, 1.29-12.22) were more likely to have a flag placed (eTable 1 in Supplement 1). Infants with government insurance were more likely to have a flag placed compared with those with private insurance (IRR, 5.49; 2.85-10.60).

**Table 5.** Flag IRR by Age Group and Race for All Encounters^a^

Age group, race	IRR (95% CI)	*P* value
**All ages**		
American Indian or Alaska Native	2.51 (0.94-6.71)	.07
Asian	1.02 (0.65-1.62)	.92
Black or African American	2.90 (1.88-4.47)	<.001
Native Hawaiian or Other Pacific Islander	1.06 (0.29-3.97)	.93
Other^b^	0.71 (0.49-1.03)	.07
Unknown or declined	2.27 (1.24-4.15)	.008
**<1 y**		
American Indian or Alaska Native	2.73 (0.51-14.57)	.24
Asian	0.33 (0.11-1.01)	.046
Black or African American	3.53 (1.80-6.91)	<.001
Native Hawaiian or Other Pacific Islander	0.36 (0.03-4.27)	.42
Other^b^	0.47 (0.24-0.90)	.02
Unknown or declined	2.21 (1.01-4.81)	.046
**1-7 y**		
American Indian or Alaska Native	0.86 (0.13-5.83)	.88
Asian	0.09 (0.01-0.74)	.03
Black or African American	2.87 (1.34-6.15)	.007
Native Hawaiian or Other Pacific Islander	2.24 (0.38-13.10)	.37
Other^b^	0.51 (0.25-1.04)	.07
Unknown or declined	0.91 (0.18-4.63)	.91
**8-12 y**		
American Indian or Alaska Native	8.75 (1.21-63.21)	.03
Asian	5.60 (2.26-13.92)	<.001
Black or African American	3.19 (1.03-9.94)	.045
Native Hawaiian or Other Pacific Islander	2.34 (0.18-30.97)	.52
Other^b^	2.03 (0.84-4.87)	.12
Unknown or declined	NC^c^	NC^c^
**13-17 y**		
American Indian or Alaska Native	2.76 (0.38-19.96)	.32
Asian	0.21 (0.04-1.09)	.06
Black or African American	1.87 (0.75-4.68)	.18
Native Hawaiian or Other Pacific Islander	NC^c^	NC^c^
Other^b^	0.72 (0.34-1.49)	.37
Unknown or declined	4.25 (1.28-14.15)	.02

Abbreviations: IRR, incident rate ratio; NC, not calculated.

^a^ White was the reference category.

^b^ Race was unspecified.

^c^ IRR could not be calculated due to small sample size.

In our sensitivity analysis limited to the first encounter for each patient, we observed similar distributions of flag placements across demographic and socioeconomic categories (eTable 2 in Supplement 1), with CPS and security flags being the most common (eTable 3 in Supplement 1). Black or African American patients and those with government insurance remained more likely to have a flag placed (eTable 4 in Supplement 1) compared with their White and privately insured counterparts.

## Discussion

In this 9-year cohort study of 55 865 pediatric encounters, 236 behavioral flags were placed, with most occurring in encounters for patients younger than 8 years. To our knowledge, this is the first study to identify inequities in behavioral flags within the inpatient setting for pediatric patients. We observed significant racial and socioeconomic disparities, with higher rates of flags placed in encounters for Black or African American patients compared with White patients and those with public compared with private insurance. This disparity persisted even when analyses were limited to encounters with infants younger than 1 year, suggesting that the placement of inappropriate behavior, security, violent behavior, and CPS hold flags may be directed toward caregivers rather than the patient. The most common flag noted was security, and while we did not have data on what led to selection of this flag vs others, this finding parallels more general social trends in our area. For example, recently the San Francisco Department of Public Health reported that 48% of the incidents of police using force at its local public hospitals and clinics involved Black patients.^14^ Similarly, a recent local study of youths experiencing behavioral health crises suggested that both Black children and adolescents were disproportionately handcuffed when using emergency medical services for behavioral health emergencies.^24^

Encounters for Black or African American patients younger than 8 years had the highest likelihood of behavioral flagging. Moreover, the density of flags in the EHRs of Black or African American patients younger than 1 year was in a group not yet reasonably capable of violent or inappropriate behavior or substance misuse. When considered alongside our finding that encounters of Black or African American patients were more frequently given CPS flags, it is possible that flags in encounters with young Black patients were a response to Black families. Our findings suggest that behavioral flags are used to mark the behavior of Black family and caregiving units as problematic. Public attitudes and beliefs about families influence not only how they are perceived but also how policies, programs, and practices are conceptualized, implemented, and evaluated.^25^ The signal about Black or African American pediatric patients from this study can be one of the data points that we use to reflect on our institution’s current conceptualization of Black families. This finding is consistent with previous studies that found higher use of security emergency response for Black patients compared with White patients.^18,19,20^ Our CPS finding is not surprising given prior work in our institution that found that Black infants and mothers were being screened more often for illicit drugs in the intensive care nursery.^22^ Since they were being screened and surveilled at a higher rate than the general population, it is not surprising that CPS would be called more often for Black families for substance use, which mirrors the literature about racial inequity in CPS engagement.^26,27^

We also found disparities in behavioral flag use in general and by flag type based on insurance status. While encounters for patients with government insurance comprised 53.5% of the study sample, they represented 80.1% of all behavioral flags. This finding parallels other work showing that publicly insured adult patients in the emergency department are at higher risk of receiving a behavioral alert and that publicly insured patients experience worse outcomes in the hospital, such as having higher rates of patient safety events.^19,28,29,30,31^ Behavioral flag inequities based on insurance status could be driven by bias, whether implicit or explicit, from health care staff, as shown in prior work studying patient socioeconomic and insurance status.^32,33,34^

Our study suggests that people who are marginalized, specifically Black people and those with lower socioeconomic status, may be more likely to be surveilled and policed both by police or security presence and via policies or procedures, such as behavioral flags, by our hospital system. The disparity in behavioral flags and security use mirrors national trends in schools and community settings; Black people in the US experience policing of their bodies at a disproportionate rate.^35,36,37,38,39,40,41,42,43,44,45^ This policing occurs both in connection with and independent of carceral systems.^7,46,47,48,49^ Hospitals and health care settings employ security officers and contract with local sheriff and police departments ostensibly to protect patients and staff; however, it is important to note that involvement of security and law enforcement in hospitals can have negative effects physically, mentally, socially, and legally.^14^ Education literature has shown that the practice of having officers in schools not only reproduces and exacerbates racial inequalities in school discipline but, importantly, worsens inequities in student entanglement with the penal system.^50^ These findings highlight the need to explore patient and staff support models that reduce or eliminate reliance on security and police.

Disparities in behavioral flagging are likely mediated by a combination of implicit and explicit biases as well as systems and structures.^51^ The racial inequities noted in our study may reflect implicit bias in patient labeling in the EHR, which has been demonstrated in prior work.^12,52,53^ As behavioral flags may cause harm by perpetuating SRD, understanding how racially minoritized pediatric patients and their families are labeled in the EHR may illuminate inequities in the hospital system that warrant deeper investigation. Because it is not the standard practice of hospitals and health care systems to consistently ask about or document SRD, work is needed to understand how and why this occurs. Research must be intersectional, meaning that it should consider demographic data in relation to the interpersonal and systemic vectors of power. Importantly, demographic categories do not indicate who is inherently more likely to cause trouble or become violent; rather, they may indicate who is being perceived as causing trouble, who is being reprimanded, and who is being policed.

We do not believe that most health care practitioners actively wish ill against Black and Indigenous people or patients who use government insurance. As Camara Phyllis Jones described, personally mediated racism need not always mean intentional racism.^51^ For example, if when encountering a patient with lower socioeconomic status or from a racial or ethnic minority group, a practitioner finds themself more on guard,^54^ that staff member might be more readily hypervigilant about boundaries, having been socialized to think certain people are more likely to transgress. That hypervigilant concern may become normalized within the system (ie, manifesting SRD). This mirrors a similar overpolicing phenomenon happening in other settings, such as graduate medical education and preschools.^55,56,57^

Our findings suggest that in the health care system, practitioners may be more vigilant and punitive in encounters with racially minoritized people and those with lower socioeconomic status. These attitudes and actions may not only escalate an emotionally charged interaction but may also exacerbate the legacy of health care distrust built on a history of mistreatment, unequal treatment, and unethical experimentation among racially minoritized groups. Our findings suggest that while behavioral flags may intend to reduce risk to health care staff, this may come at the cost of increasing risk of harm to the patient and/or their family (eg, the patient’s risk of being removed from their family of origin, being medically restrained, having an altercation with police, or having delays in care). More research is needed to discern the outcomes of behavioral flag use for pediatric patients and how health care systems can effectively reduce inequities in behavioral flag applications.

While our study did not allow us to understand why inequities in flagged encounters were so pronounced, given how intertwined beliefs and expectations are with actions, how our findings might be related to how dominant narratives about racial and ethnic groups remain in our health care system may be a subject for future research. Our system has begun examining each newly placed flag to evaluate its purpose and appropriateness via committee and by limiting the duration of the flag in the EHR to a maximum of 3 years to more robustly interrogate the context surrounding the flag application. Also, we have developed a new behavioral de-escalation support team (called Code CARE at our institution) that trains staff in antiracist praxis and trauma-informed care to add an additional resource for families and practitioners when tense moments arise during hospitalization. Important next steps will be to advocate for consistent documentation and analysis of social determinants of health and SRD and to more thoroughly examine the context surrounding flag assignment. To do this, we must interrogate not only biases against marginalized groups but also the values and norms that inform the rules and regulations that minoritized people are penalized for failing to uphold (ie, to practice cultural humility).

### Limitations

Our study has several limitations. The data we used, like most data collected by health care systems about race and ethnicity, are variable, nonspecific, and influenced by context. It is well established that race is a social (rather than a biologic) construct. Racial and ethnic categories have changed over time and will continue to do so with the evolution of various social movements and projects. The boundaries of racial categories have never been fixed and never will be, which makes studying the effects of race (or more specifically, of being socially racialized into a particular category within our society) using only a limited number of fixed categories not only difficult but inherently insufficient. In our study, there were only 6 racial categories from which to choose (American Indian or Alaska Native, Asian, Black or African American, Native Hawaiian or Other Pacific Islander, White, and other), rather than the 63 possible permutations of racial and ethnic identity available in the most recent US census. Furthermore, in our system, parents or caregivers select the racial identification for the child, which may or may not be concordant with a specific caregiver. Thus, continuing to solely capture the social construct of race in our health care records provides an insufficient proxy for inequities that exist and are mediated via racism and other forms of discrimination and goes against existing best practices.^58,59,60^ Furthermore, it reifies the biologic representation of race, which medicine has perpetuated for too long. To truly address these inequities, it is essential to incorporate a broader range of factors, such as socioeconomic status, neighborhood conditions, and access to resources, and markers of structural inequities. Of note, our hospital system has begun to collect data on other social determinants of health, such as food, transportation, and financial security, that can be used in future studies to further explore inequities.

Another major limitation of our study is that we were unable to tell when disparities in flag use were due to differences in documentation (eg, being more likely to document a security flag in certain populations) or actions (eg, being more likely to call security to the rooms of certain populations). We did not have information on drug screening or whether any of the patients had psychiatric holds for dysregulated behavior. Additionally, the lack of flag specificity beyond broad categories diminished the individual meanings; the vagueness of the flags did not allow for further examination of what was happening when a flag was placed or how the flag influenced practitioners’ actions after it was placed. For example, we could not distinguish when hospital security or police were physically present or whether or when restraints or force were used based on behavioral flags alone.

Additionally, the small sample size of American Indian or Alaska Native patients meant that the differences in application of behavioral flags were not statistically significant. However, rates of flag use were disproportionate. The way that flagging and other risk interventions are used for Indigenous patients requires more consideration and analysis.

## Conclusions

This cohort study demonstrated that in an inpatient pediatric setting, health care practitioners disproportionately applied negative patient labels to racially and socioeconomically marginalized patients and families. Additionally, we found a concentration of this pattern among Black or African American patients younger than 1 year, suggesting that our health care system’s current practice of flag application may have more to do with Black families than the actions of pediatric patients. These findings suggest that efforts are needed to eliminate unequal treatment of pediatric patients and their families by working toward change at our own institutions and through national policies that will ensure that hospitals are safe spaces for all in need of care.

## Notes

Supplement 1. **eTable 1.** Flag Incident Rate Ratio by Race, Gender, and Insurance for Patients Younger Than 1 Year for All Encounters, Unadjusted and Adjusted for All Variables of Interest **eTable 2.** Demographic and Encounter Characteristics by Presence of Flag, Limited to First Encounter **eTable 3.** Type of Flags by Racial Groups, Limited to First Encounter **eTable 4.** Flag Incident Rate Ratio by Race, Gender, and Insurance Among All Patients Younger Than 18 Years, Limited to First Encounter and Unadjusted and Adjusted for All Variables of Interest

Supplement 2.
Data Sharing Statement
