# Supplementary material for: Inequities in the Application of Behavioral Flags for Hospitalized Pediatric Patients
Source: JAMA Netw Open. 2025 Feb 20;8(2):e2461079. doi: 10.1001/jamanetworkopen.2024.61079 (PMC11843364; doi:10.1001/jamanetworkopen.2024.61079)
Supplement: Supplement 1. — eTable 1. Flag Incident Rate Ratio by Race, Gender, and Insurance for Patients Younger Than 1 Year for All Encounters, Unadjusted and Adjusted for All Variables of Interest eTable 2. Demographic and Encounter Characteristics by Presence of Flag, Limited to First Encounter eTable 3. Type of Flags by Racial Groups, Limited to First Encounter eTable 4. Flag Incident Rate Ratio by Race, Gender, and Insurance Among All Patients Younger Than 18 Years, Limited to First Encounter and Unadjusted and Adjusted for All Variables of Interest [file jamanetwopen-e2461079-s001.pdf]

Supplementary Online Content

Edwell A, Huang JX, Bongiovanni T, Pantell M. Inequities in the application of behavioral flags for hospitalized pediatric patients. *JAMA Netw Open*. 2025;8(2):e2461079. doi:10.1001/jamanetworkopen.2024.61079

eTable 1. Flag Incident Rate Ratio by Race, Gender, and Insurance for Patients Younger Than 1 Year for All Encounters, Unadjusted and Adjusted for All Variables of Interest
eTable 2. Demographic and Encounter Characteristics by Presence of Flag, Limited to First Encounter
eTable 3. Type of Flags by Racial Groups, Limited to First Encounter
eTable 4. Flag Incident Rate Ratio by Race, Gender, and Insurance Among All Patients Younger Than 18 Years, Limited to First Encounter and Unadjusted and Adjusted for All Variables of Interest

This supplementary material has been provided by the authors to give readers additional information about their work.

© 2025 Edwell A et al. *JAMA Network Open*.

eTable 1: Flag incident rate ratio by race, gender, and insurance for patients <1 year old for all encounters, unadjusted and adjusted for all variables of interest

	Unadjusted		Adjusted	
**Variables**	**IRR [95% CI]**	**p-value**	**IRR [95% CI]**	**p-value**
**Race**				
Asian	0.33 [0.11, 1.01]	0.05	0.36 [0.11, 1.13]	0.08
Black/African American	3.53 [1.80, 6.91]	<0.001	1.96 [1.00, 3.82]	0.05
Native American/Alaska Native	2.73 [0.51, 14.57]	0.24	1.75 [0.35, 8.72]	0.49
Native Hawaiian/Other Pacific Islander	0.36 [0.03, 4.27]	0.42	0.26 [0.02, 3.14]	0.29
Other	0.47 [0.24, 0.90]	0.07	0.50 [0.23, 1.10]	0.08
Unknown/Declined	2.21 [1.01, 4.81]	0.05	3.97 [1.29, 12.22]	0.02
White	[ref]		[ref]	
**Ethnicity**				
Not Hispanic/Latino	[ref]		[ref]	
Hispanic/Latino	0.41 [0.22, 0.74]	<0.01	0.45 [0.22, 0.94]	0.03
Unknown/Declined	1.00 [0.42, 2.35]	0.99	0.29 [0.08, 1.02]	0.05
**Insurance**				
Private	[ref]		[ref]	
Government	5.33 [2.81, 10.14]	<0.001	5.49 [2.85, 10.60]	<0.001

Abbreviations: CI = Confidence Interval, IRR = Incident rate ratio, ref = reference

© 2025 Edwell A et al. *JAMA Network Open*.

eTable 2: Demographic and encounter characteristics by presence of flag, limited to first encounter

Characteristics	Total Cohort N (%)	Flag, N = 138 N (%)	No Flag, N = 32,799 N (%)	*p*-value
**Age**				<0.001
<1 yr	11,899 (36.1)	73 (52.9)	11,826 (39.1)	
1 – 7 yr	8,742 (26.5)	20 (14.5)	8,722 (26.6)	
8 – 12 yr	4,967 (15.1)	15 (11.0)	4,952 (15.1)	
13 – 17 yr	7,329 (22.2)	30 (21.7)	7,299 (22.3)	
**Sex**				0.78
Male	17,203 (52.2)	68 (49.3)	17,135 (52.2)	
Female	15,729 (47.8)	70 (50.7)	15,659 (47.7)	
Unknown	5 (0.0)	0 (0)	5 (0)	
**Race**				<0.001
Asian	3,969 (12.1)	5 (3.6)	3,964 (12.1)	
Black/ African American	2,787 (8.5)	37 (26.8)	2,750 (8.4)	
Native American/ Alaska Native	276 (0.8)	4 (2.9)	272 (0.8)	
Native Hawaiian/other Pacific Islander	356 (1.1)	1 (0.7)	355 (1.1)	
White	12,464 (37.8)	41 (29.7)	12,423 (37.9)	
Other	11,073 (33.6)	35 (25.4)	11,038 (33.7)	
Unknown/Declined	2,012 (6.1)	15 (10.9)	1,997 (6.1)	
**Ethnicity**				0.04
Hispanic/Latino	10,727 (32.6)	36 (26.1)	10,691 (32.6)	
Not Hispanic/Latino	20,113 (61.1)	87 (63.0)	20,026 (61.1)	
Unknown	2,097 (6.4)	17 (7.2)	2,563 (4.6)	
**Language**				0.005
English	27,569 (83.7)	132 (95.7)	27,437 (83.7)	
Spanish	4,354 (13.2)	5 (3.6)	4,349 (13.3)	
Chinese	296 (0.9)	0 (0)	296 (0.9)	
Other	578 (1.8)	1 (0.7)	577 (1.8)	
Unknown/Declined	140 (0.4)	0 (0)	140 (0.4)	
**Insurance**				
Government	16,850 (51.2)	112 (81.2)	16,738 (51.0)	
Private	15,689 (47.6)	26 (18.8)	15,663 (47.8)	
Other	398 (1.2)	0 (0)	398 (1.2)	<0.001

© 2025 Edwell A et al. *JAMA Network Open*.

eTable 3: Type of flags by racial groups, limited to first encounter

	Racial groups, N (%)							
**Types of Flags** (%)	**Asian**	**Black/ African American**	**Native American/ Alaska Native**	**Native Hawaiian/ Other Pacific Islander**	**Other**	**Unknown/ Declined**	**White**	***p*-value**
N = 138	N=5	N=37	N=4	N=1	N=35	N=15	N=41	
**Inappropriate behavior** (5)	1 (2)	2 (5)	0 (0)	0 (0)	1 (3)	0 (0)	3 (7)	0.58
**Security** (41)	2 (4)	9 (24)	2 (50)	1 (100)	17 (49)	6 (40)	20 (49)	0.03
**Witnessed Substance Use** (1)	0 (0)	0 (0)	0 (0)	0 (0)	0 (0)	0 (0)	2 (5)	0.77
**Violent behavior** (10)	1 (2)	7 (19)	0 (0)	0 (0)	3 (8)	0 (0)	3 (7)	<0.001
**CPS hold** (42)	1 (2)	19 (51)	2 (50)	0 (0)	14 (40)	9 (60)	13 (32)	<0.001

© 2025 Edwell A et al. *JAMA Network Open*.

eTable 4. Flag incident rate ratio by race, gender, and insurance among all patients < 18 years old limited to first encounter, unadjusted and adjusted for all variables of interest

	Unadjusted		Adjusted	
**Variables**	**IRR [95% CI]**	***p*-value**	**IRR [95% CI]**	***p*-value**
**Race**				
Asian	0.29 [0.11, 0.77]	0.01	0.29 [0.11, 0.78]	0.01
Black/African American	3.53 [21.11, 5.92]	<0.001	2.14 [1.25, 3.64]	0.005
Native American/Alaska Native	3.04 [0.81, 11.48]	0.10	2.15 [0.58, 7.98]	0.26
Native Hawaiian/Other Pacific Islander	0.37 [0.04, 3.79]	0.41	0.29 [0.28, 3.04]	0.30
Other	0.84 [0.53, 1.35]	0.48	0.68 [0.39, 1.18]	0.17
Unknown/Declined	2.38 [1.25, 4.51]	0.008	1.83 [0.68, 4.93]	0.24
White	[ref]		[ref]	
**Ethnicity**				
Not Hispanic/Latino	[ref]		[ref]	
Hispanic/Latino	0.74 [0.49, 1.14]	0.18	0.72 [0.43, 1.23]	0.23
Unknown/Declined	1.68 [0.89, 3.17]	0.11	1.08 [0.39, 2.95]	0.88
**Insurance**				
Private	[ref]		[ref]	
Government	3.90 [2.50, 6.09]	<0.001	3.78 [2.37, 6.05]	<0.001

Abbreviations: IRR = Incident rate ratio, CI = Confidence Interval.

© 2025 Edwell A et al. *JAMA Network Open*.
