# Supplementary material for: Inequities in the Application of Behavioral Flags for Hospitalized Pediatric Patients
Source: JAMA Netw Open. 2025 Feb 20;8(2):e2461079. doi: 10.1001/jamanetworkopen.2024.61079 (PMC11843364; doi:10.1001/jamanetworkopen.2024.61079)
Supplement: Supplement 2. — Data Sharing Statement [file jamanetwopen-e2461079-s002.pdf]

Data Sharing Statement

Edwell. Inequities in the Application of Behavioral Flags for Hospitalized Pediatric Patients. *JAMA Netw Open*. Published February 20, 2025. doi:10.1001/jamanetworkopen.2024.61079

Data
Data available: Yes
Data types: Deidentified participant data
How to access data: matt.pantell@ucsf.edu
When available: With publication

Supporting Documents
Document types: None

Additional Information
Who can access the data: Researchers whose proposed use has been proposed.
Types of analyses: Analyses that investigate demographic differences in the EHR.
Mechanisms of data availability: With a signed data access agreement.
